# Suppression of Rapidly Progressive Mouse Glomerulonephritis with the Non-Steroidal Mineralocorticoid Receptor Antagonist BR-4628

**DOI:** 10.1371/journal.pone.0145666

**Published:** 2015-12-23

**Authors:** Frank Y. Ma, Yingjie Han, David J. Nikolic-Paterson, Peter Kolkhof, Greg H. Tesch

**Affiliations:** 1 Department of Nephrology, Monash Medical Centre, Clayton, Victoria, Australia; 2 Department of Medicine, Monash University, Clayton, Victoria, Australia; 3 Department of Cardiology Research, Global Drug Discovery, Bayer HealthCare, Wuppertal, Germany; Emory University, UNITED STATES

## Abstract

**Background/Aim:**

Steroidal mineralocorticoid receptor antagonists (MRAs) are effective in the treatment of kidney disease; however, the side effect of hyperkalaemia, particularly in the context of renal impairment, is a major limitation to their clinical use. Recently developed non-steroidal MRAs have distinct characteristics suggesting that they may be superior to steroidal MRAs. Therefore, we explored the benefits of a non-steroidal MRA in a model of rapidly progressive glomerulonephritis.

**Methods:**

Accelerated anti-glomerular basement membrane (GBM) glomerulonephritis was induced in groups of C57BL/6J mice which received no treatment, vehicle or a non-steroidal MRA (BR-4628, 5mg/kg/bid) from day 0 until being killed on day 15 of disease. Mice were examined for renal injury.

**Results:**

Mice with anti-GBM glomerulonephritis which received no treatment or vehicle developed similar disease with severe albuminuria, impaired renal function, glomerular tuft damage and crescents in 40% of glomeruli. In comparison, mice which received BR-4628 displayed similar albuminuria, but had improved renal function, reduced severity of glomerular tuft lesions and a 50% reduction in crescents. The protection seen in BR-4628 treated mice was associated with a marked reduction in glomerular macrophages and T-cells and reduced kidney gene expression of proinflammatory (CCL2, TNF-*α*, IFN-γ) and profibrotic molecules (collagen I, fibronectin). In addition, treatment with BR-4626 did not cause hyperkalaemia or increase urine Na+/K+ excretion (a marker of tubular dysfunction).

**Conclusions:**

The non-steroidal MRA (BR-4628) provided substantial suppression of mouse crescentic glomerulonephritis without causing tubular dysfunction. This finding warrants further investigation of non-steroidal MRAs as a therapy for inflammatory kidney diseases.

## Introduction

Steroid-based mineralocorticoid receptor (MR) antagonists (spironolactone and eplerenone) provide protection against kidney and cardiovascular disease through both local tissue effects and by reducing hypertension.[1,2] Steroidal MRAs can also inhibit kidney injury in animal models of glomerulonephritis and diabetic nephropathy independent of blood pressure effects.[3–8] In addition, steroidal MRAs have consistently provided added protection in glomerulonephritis and diabetic nephropathy in clinical trials when used in conjunction with renin-angiotensin system (RAS) blockade (the current standard therapy), which supports their use as an adjunct therapy.[9–11]

Despite their therapeutic benefits, steroidal MRAs have drawbacks which limit their clinical use. In addition to binding to the MR, spironolactone binds to progesterone and androgen receptors leading to adverse progestational and anti-androgenic effects (including gynecomastia, breast tenderness, impotence and menstrual irregularities).[12] In comparison, eplerenone is more selective, but has weaker affinity for binding MR and is less potent than spironolactone.[12] Eplerenone is metabolised by the ubiquitous cytochrome (CYP) 3A4 and drugs or antibiotics that inhibit CYP3A4 can precipitate eplerenone toxicity.[12] A further downside of steroidal MRA therapy is that it can cause hyperkalaemia in patients, which is a major clinical concern, particularly in the context of renal impairment, and necessitates withdrawal of this treatment.[9] This problem arises because steroidal MRAs inhibit aldosterone-based activation of ion channels in tubular epithelial cells which is essential for sodium and potassium homeostasis. Blocking this pathway elevates potassium levels, which is exacerbated during RAS blockade. Therefore, it is desirable to develop a therapy which can inhibit pathological MR signaling while having a minimal effect on potassium homeostasis.

Recent high throughput screening analysis has identified dihydropyridine and pyrazoline derivatives that can bind to MR and inhibit MR signalling responses. Some of these non-steroidal compounds are highly selective for MR and provide similar or better protection than steroidal MRAs in rodent models of renal injury induced by mineralocorticoid infusion and hypertension.[13–15] These non-steroidal MRAs are also reported to have reduced adverse side effects compared to steroidal MRAs.

Previously, we and others have identified a pathogenic role for the MR in the development of mouse antibody-dependent glomerulonephritis.[3,5] Conditional gene deletion studies have shown that renal injury in mice with anti-glomerular basement membrane (GBM) glomerulonephritis is mediated by MR signaling in macrophages,[5] Furthermore, kidney damage in this model does not involve hypertension,[16] which can be MR-dependent. This has prompted us to examine whether treatment with a non-steroidal MR antagonist (BR4628)[17] can specifically inhibit glomerular injury caused by macrophage MR signaling, as a proof of principle. Given that macrophage-mediated injury is known to play a key role in chronic kidney diseases (CKD), the findings of this study may provide insight into the potential of non-steroidal MRA therapy in CKD patients. In addition, we identified whether BR-4628 treatment could provide protection against glomerulonephritis without causing tubular dysfunction that results in elevated Na+/K+ excretion, indicating a loss of potassium homeostasis.

## Methods

### Mineralocorticoid Receptor Blockade

BR-4628 (supplied by Bayer Pharma AG) is a recently developed non-steroidal MR antagonist derived from the structure of dyhydropyridines.[18] The dosage of BR-4628 used in this study is based on its affinity for MR and previous research showing this dosage can reduce renal injury in a rat model of mineralocorticoid-induced hypertension. [13]

### Animal Model

Groups of 12 week old female C57BL/6 mice (n = 8) were pre-immunised subcutaneously with 1mg sheep IgG in Freund’s complete adjuvant. Four days later, glomerulonephritis was induced by intravenous injection of sheep anti-mouse GBM serum (10μl/g).[5] Groups of mice with glomerulonephritis received either BR-4628 (5mg/kg/bid in vehicle) or vehicle (1ml/kg, Solutol 50%, 45% water, 5% ethanol, v/v/v) or water (1ml/kg, untreated controls). Treatment by oral gavage commenced 2 hours prior to injection of anti-GBM serum on day 0 and continued twice daily until day 15 when mice were given anaesthesia (ketamine 120mg/kg and xylazine 5mg/kg by intraperitoneal injection) and killed by cervical dislocation. Additional groups of 8 age-matched female mice without disease received either vehicle or BR-4628 for 15 days before being killed as normal controls. Experimental mice were maintained under standard animal house conditions in a conventional animal facility. All animal experiments were approved by the Monash Medical Centre Animal Ethics Committee (Approval Number: MMCB 2014/03) and were conducted in strict accordance with the Australian Code of Practice for the Care and Use of Animals for Scientific Purposes, 8^th^ edition (2013).

### Hematology and Biochemical Analysis

Urine was collected from mice housed in metabolic cages for 6 hours (0900–1500) before experimentation and at days 1, 7 and 14 of disease. At day 15, heparinised blood and serum were collected from anaesthetised mice. Urine creatinine levels were determined by the Jaffe rate reaction method. ELISAs were used to measure urine levels of albumin (Bethyl Laboratories, Montgomery, TX) and serum levels of cystatin-C (Enzo Life Sciences, Farmingdale, NY, USA). Levels of K+ or Na+ in plasma or urine were determined by indirect potentiometry using ion selective electrodes.

### Histology Analysis

Formalin-fixed kidney sections (2μm) were stained with periodic acid Schiff’s (PAS) reagent to identify kidney structure, including glomerular tuft lesions (capillary thrombosis, mesangial matrix accumulation) and glomerular crescent formation. Hematoxylin staining was used to distinguish cell nuclei. The severity of glomerular tuft lesions was assessed in at least 50 glomerular cross-sections in each animal by a semi-quantitative score: 0 = no thrombosis, <10% mesangial matrix in glomerular tuft; 1 = 10–25% of glomerular tuft with thrombosis and/or matrix; 2 = 25–50% of glomerular tuft with thrombosis and/or matrix; 3 = 50–75% glomerular tuft with thrombosis and/or matrix; 4 = 75–100% glomerular tuft with thrombosis or matrix. The percentage of glomeruli with crescent formation was assessed in at least 50 glomerular cross-sections in each animal. All scoring was performed on blinded slides.

### Immunostaining Analysis

Immunoperoxidase staining for leukocytes was performed on 2% paraformaldehyde-lysine-periodate-fixed kidney cryostat sections (4μm) using anti-CD68 (FA-11, Serotec, Oxford, UK) and anti-CD3 (KT3, Abcam) antibodies according to established protocols.[5] To assist leukocyte counting, cell nuclei were weakly stained with hematoxylin. The number of macrophages (CD68+) and T-cells (CD3+) was assessed in 20 glomeruli per kidney section. Immunoperoxidase staining for apoptotic cells was performed on methylcarn-fixed paraffin-embedded kidney sections (4μm) using an antibody to activated caspase-3.[5] The number of apoptotic cells (activated caspase-3+) in cortical tubules was assessed in each animal and recorded as apoptotic cells per 1000 tubule cross-sections (tcs). All scoring was performed on blinded slides.

### Real-time PCR

Total RNA was extracted from whole kidney using Trizol (Invitrogen) and reverse transcribed with random primers using the Superscript First-Strand Synthesis kit (Invitrogen). Real-time PCR analysis was performed as previously described.[19] The Taqman probe and primer sequences are listed in Table A in S1 Appendix.

### Statistical Analysis

Statistical differences between two groups were analysed by t-test and differences between multiple groups were assessed by ANOVA with post hoc analysis using Tukey’s multiple comparison test. Data was analysed using Graph Prism 6.0 (GraphPad Software, San Diego, CA) and was recorded as the mean ± standard error with p<0.05 defined as significant.

## Results

### BR-4628 protects mice from declining renal function in glomerulonephritis

**Fig 1:**
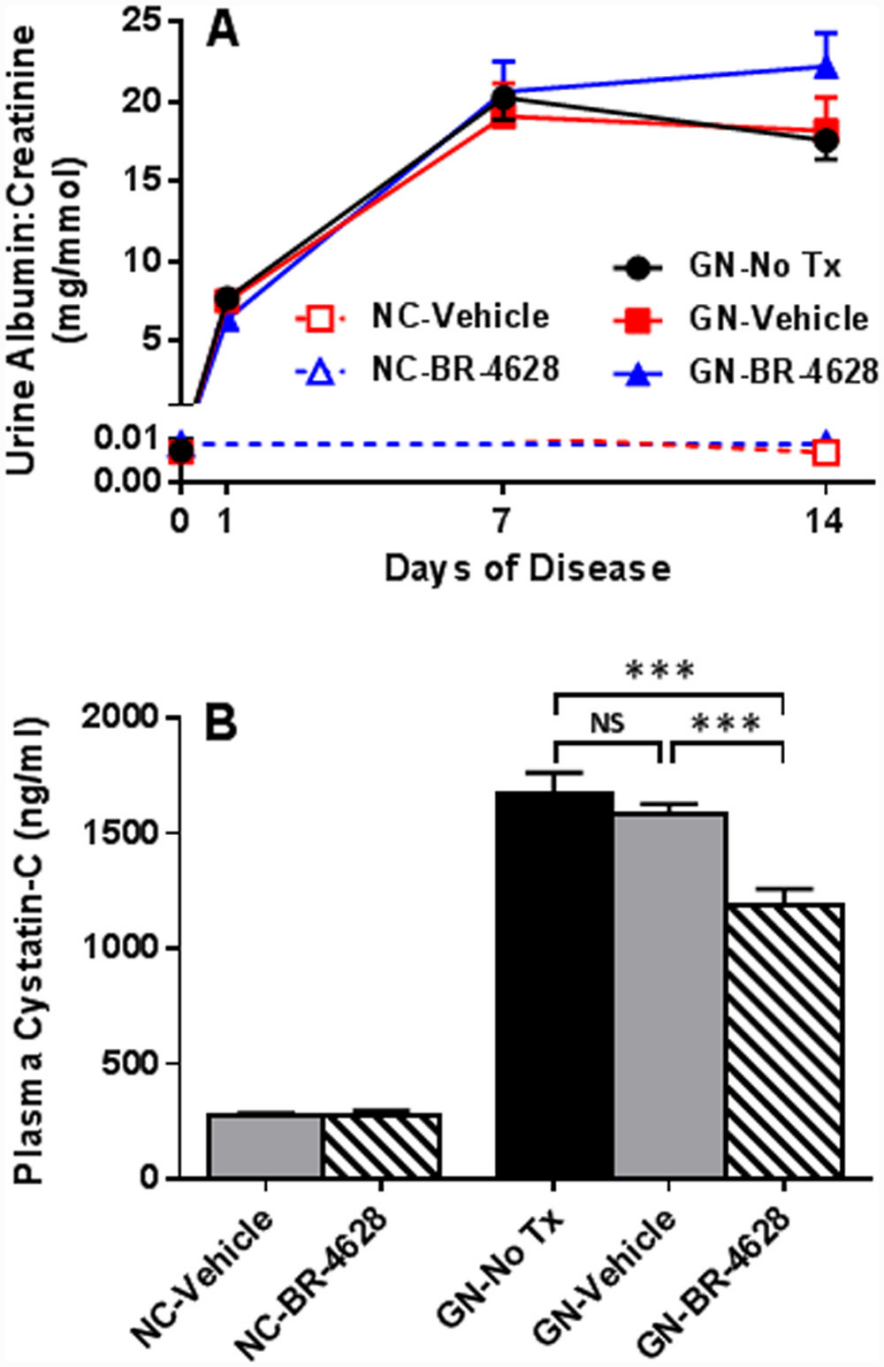
BR-4628 protects mice from loss of renal function in anti-GBM glomerulonephritis. (A) Compared to normal control (NC) mice given vehicle (red unfilled squares) or BR-4628 (blue unfilled triangles), urine albumin excretion was massively increased at days 1, 7 and 14 of glomerulonephritis (GN) in mice receiving no treatment (No Tx, black filled circles) or vehicle (red filled squares), and these albuminuria levels were unaffected by treatment with BR-4628 (blue filled triangles). (B) At day 15 of glomerulonephritis, there was a 6-fold increase in serum levels of cystatin-C in mice receiving no treatment or vehicle, which was reduced by 30% in mice treated with BR-4628. Data = mean ± SEM; n = 8. ***p<0.001.

Compared to normal controls, there was a 1000-fold increase in the urine albumin excretion at day 1 of glomerulonephritis, which increased 3-fold further at day 7 of disease and then remained stable between days 7 and 14 of disease (Fig 1A). There was no difference in urine albumin excretion between groups with glomerulonephritis (untreated, vehicle or BR-4628 treated) at any of the time-points examined (days 1, 7 and 14). Albuminuria was not observed in normal control (NC) mice which received either vehicle or BR-4628 (Fig 1A).

Development of glomerulonephritis was associated with a significant loss of renal function as demonstrated by a 6-fold increase in the plasma levels of cystatin-C at day 15 of disease in untreated and vehicle-treated groups, compared to normal controls (Fig 1B). In comparison, mice with glomerulonephritis which received BR-4628 showed a 30% reduction in plasma levels of cystatin-C at day 15 (Fig 1B), demonstrating that BR-4628 significantly improves renal function. In the absence of glomerulonephritis, renal function remained normal in groups treated with vehicle or BR-4628 (Fig 1B).

### BR-4628 inhibits glomerular and tubular damage in glomerulonephritis

**Fig 2:**
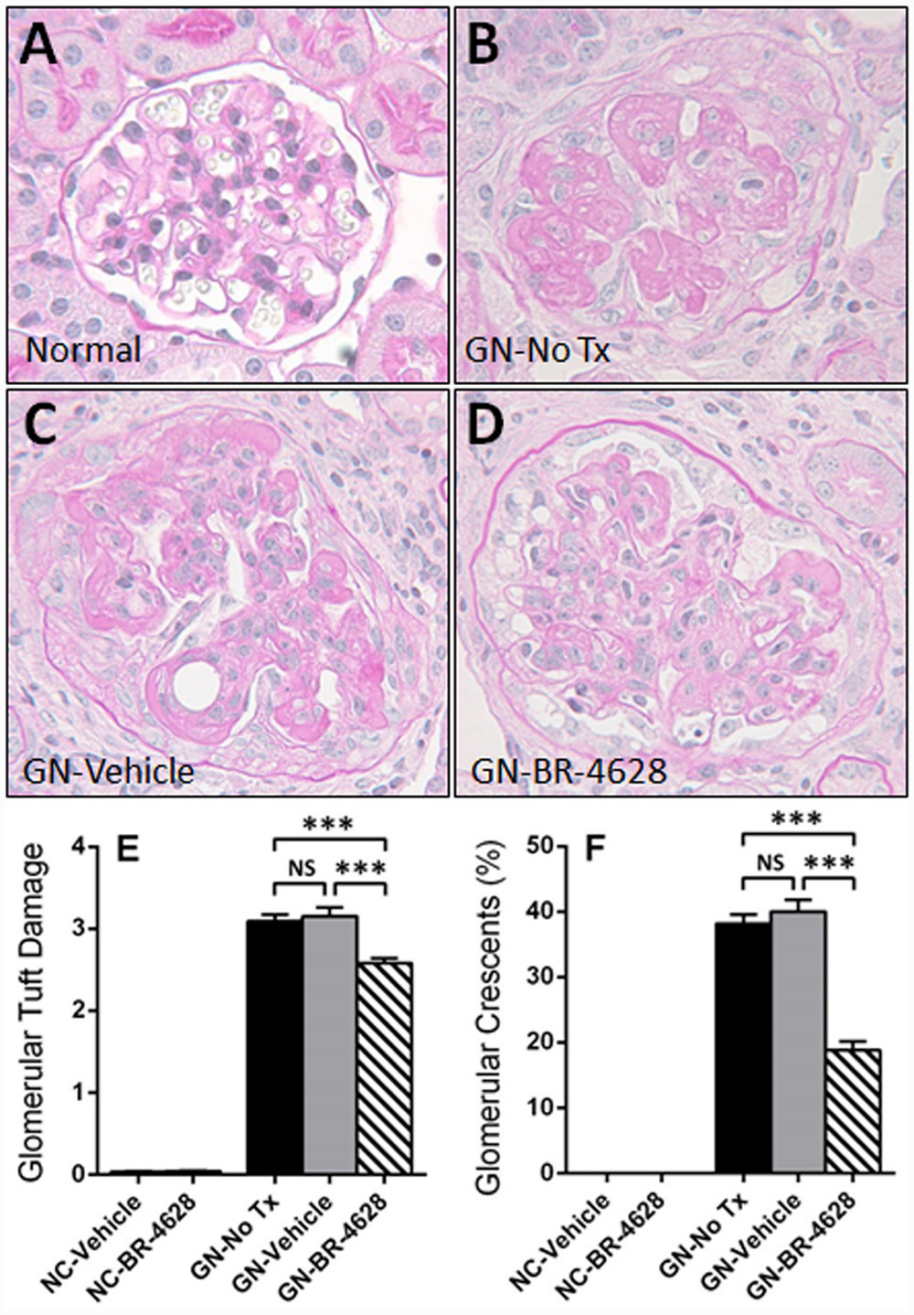
BR-4628 reduces glomerular lesions in anti-GBM glomerulonephritis. Histological staining with PAS and hematoxylin shows the kidney structure of (A) a normal mouse. In comparison, there is severe damage to glomeruli (capillary thrombosis, glomerulosclerosis, crescent formation) at day 15 of glomerulonephritis in (B) a mouse receiving no treatment and (C) a mouse receiving vehicle, which is attenuated in (D) a mouse treated with BR-4628. Graphs show (E) the severity score of glomerular tuft damage (0–4+) and (F) the percentage of glomeruli with crescents in kidneys from normal control (NC) mice and mice with glomerulonephritis (GN) receiving no treatment (No Tx), vehicle or BR-4628. Magnification: (A-D) x400. Graph Data = mean ± SEM; n = 8. *p<0.05, ***p<0.001, NS = not significant.

**Fig 3:**
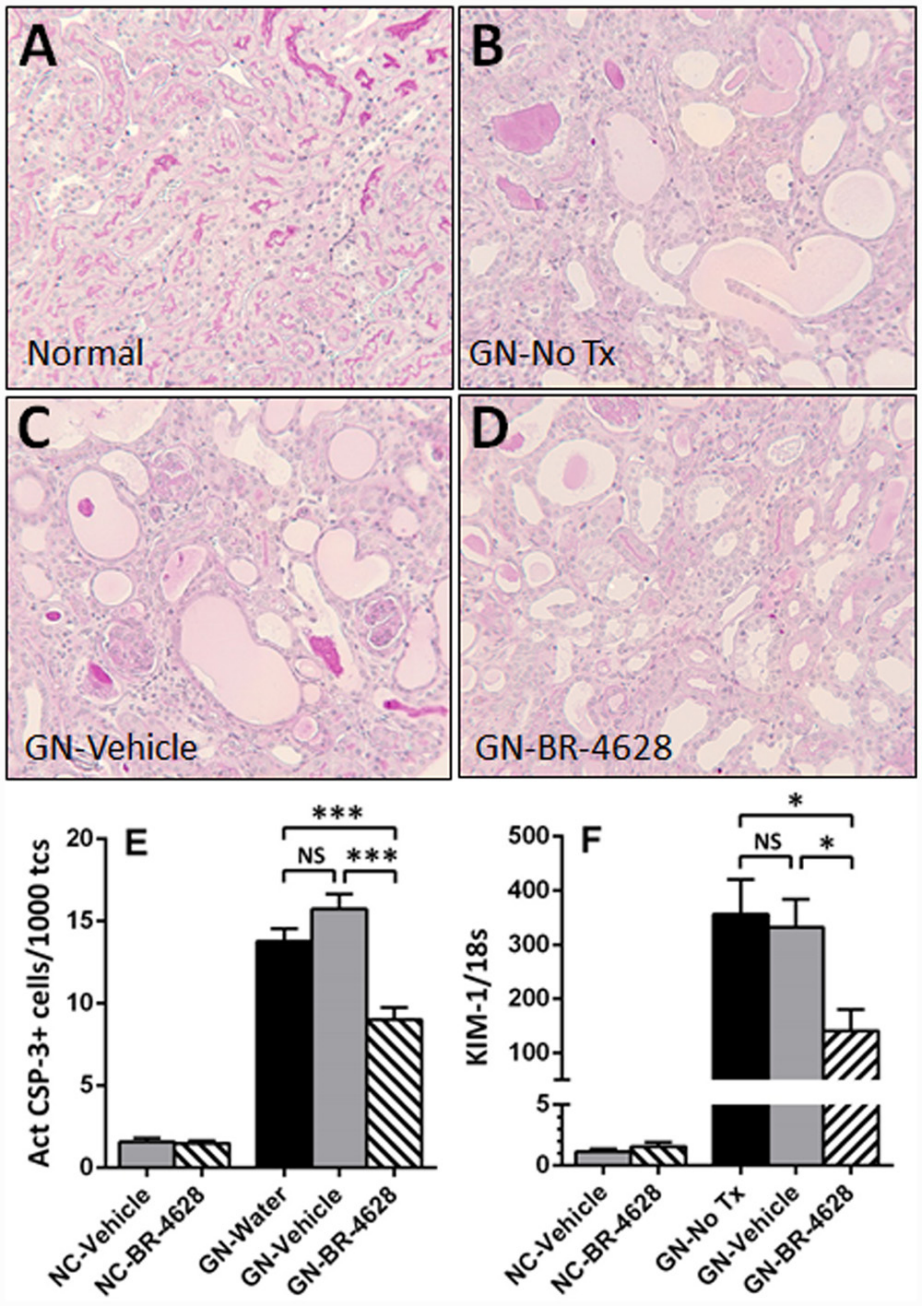
BR-4628 reduces tubular damage in anti-GBM glomerulonephritis. Histological staining with PAS and hematoxylin shows the structure of kidney tubules in (A) a normal mouse. In comparison, there is severe damage to tubules (dilation, atrophy, cell loss) at day 15 of glomerulonephritis in (B) a mouse receiving no treatment and (C) a mouse receiving vehicle, which is attenuated in (D) a mouse treated with BR-4628. Graphs show (E) the number of activated caspase-3+ apoptotic cells in tubules in the kidney cortex and (F) the gene expression of kidney injury molecule-1 (KIM-1) in kidneys from normal control (NC) mice and mice with glomerulonephritis (GN) receiving no treatment (No Tx), vehicle or BR-4628. Magnification: (A-D) x400. Graph Data = mean ± SEM; n = 8. *p<0.05, ***p<0.001, NS = not significant.

At day 15 of glomerulonephritis, untreated and vehicle-treated mice had severe histological glomerular damage which included the development of thrombosis and sclerosis in the glomerular tuft, and crescents in 40% of glomeruli (Fig 2B, 2C, 2E and 2F). In comparison, mice treated with BR-4628 had less severe glomerular tuft damage and only 19% of glomeruli with crescents (Fig 2D, 2E and 2F). Extensive histological tubular damage (dilation, atrophy, cell loss) was also found in the renal cortex of untreated and vehicle-treated mice at day 15 of disease (Fig 3B and 3C), but appeared less in mice receiving BR-4628 (Fig 3D). The extent of tubular injury in untreated and vehicle-treated mice with glomerulonephritis was demonstrated by a 10-fold increase in the number of apoptotic (activated caspase-3+) tubular cells and a 300-fold increase in gene expression of kidney injury molecule-1 (KIM-1), which were both significantly reduced with BR-4628 treatment (Fig 3E and 3F).

### BR-4628 suppresses the development of renal fibrosis in glomerulonephritis

**Fig 4:**
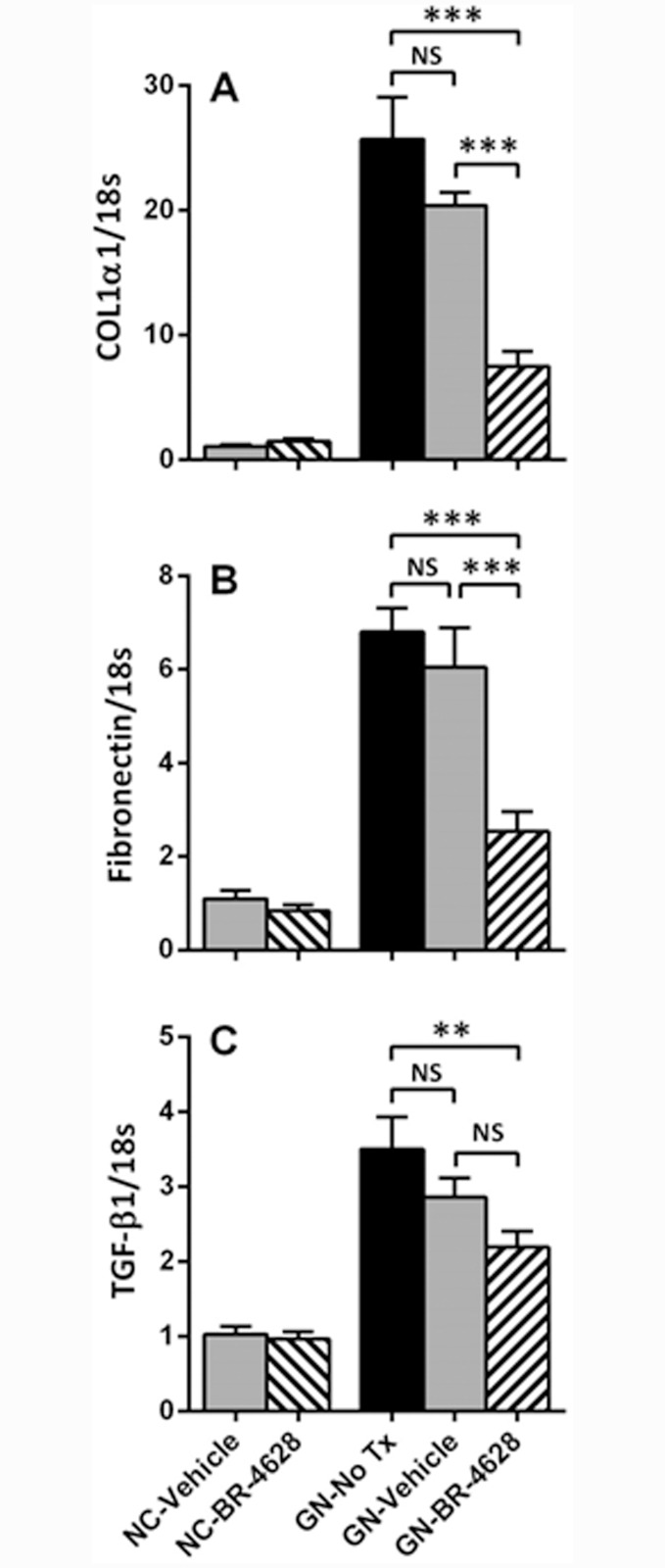
BR-4628 reduces kidney gene expression of pro-fibrotic molecules in anti-GBM glomerulonephritis. RT-PCR analysis showed that in comparison to normal control (NC) mice, there was a marked increase in the kidney mRNA levels of (A) collagen 1 (α1 chain), (B) fibronectin and (C) TGF-β1 at day 15 of glomerulonephritis in mice receiving no treatment (No Tx), which was similar to mice receiving vehicle. Treatment with BR-4628 reduced the kidney mRNA level of each of these profibrotic molecules. Data = mean ± SEM; n = 8. **p<0.01, ***p<0.001, NS = not significant.

Real-time PCR analysis of whole kidney identified increases in the gene expression of collagen I (25-fold), fibronectin (7-fold) and TGF-β1 (3.5-fold) in untreated mice with glomerulonephritis, which was similar in vehicle-treated mice (Fig 4). BR-4628 treatment reduced the gene expression of collagen 1 and fibronectin by 60–70% in diseased kidneys, compared to vehicle and no treatment (Fig 4A and 4B). BR-4628 also reduced the gene expression of TGF-β1 in diseased kidneys, when compared to untreated mice (Fig 4C).

### BR-4628 reduces renal inflammation in glomerulonephritis

**Fig 5:**
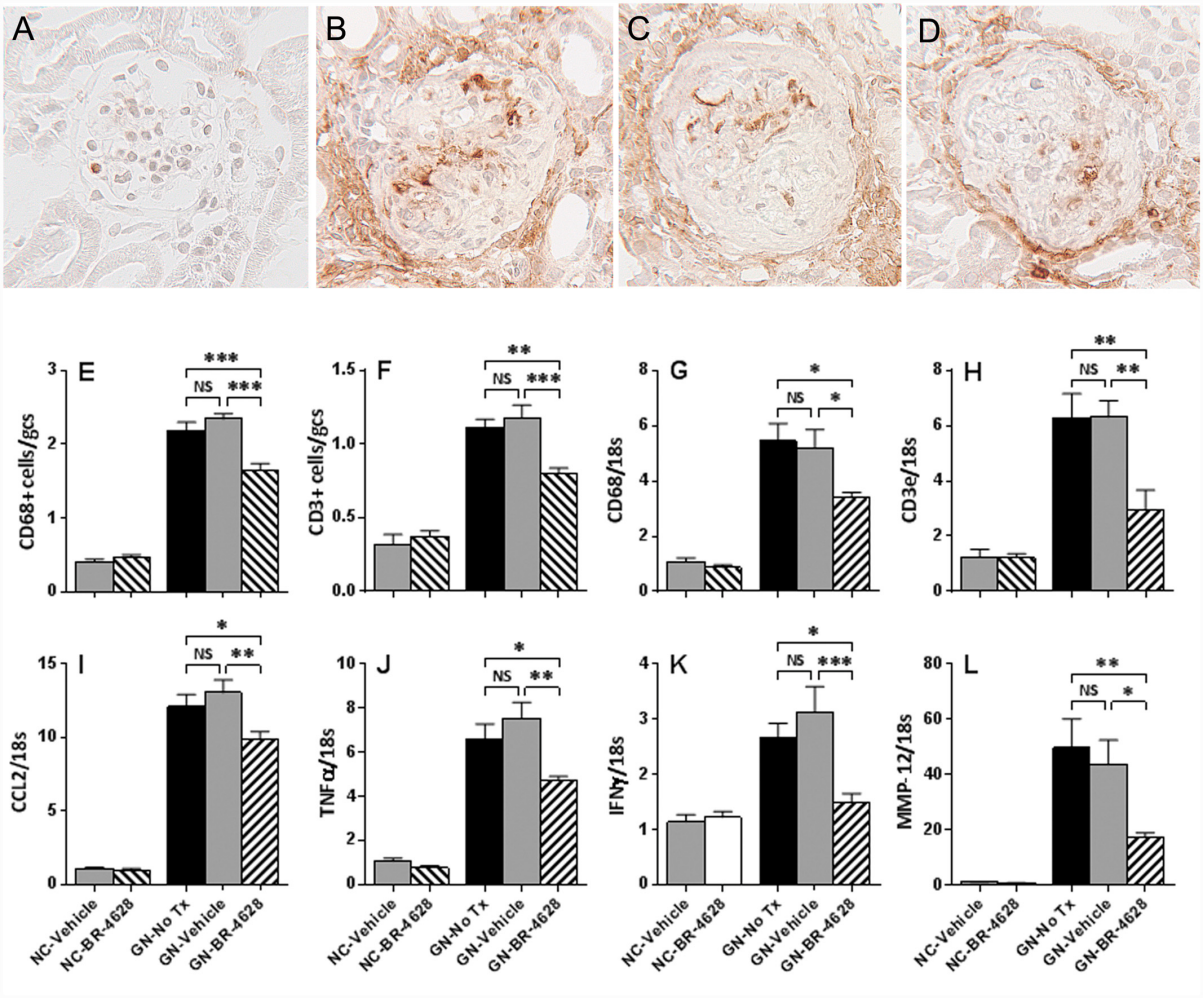
BR-4628 reduces inflammation in anti-GBM glomerulonephritis. Immunostaining of a CD68+ macrophage (brown) was seen in a minor proportion of (A) the glomeruli of normal control (NC) mice. In comparison, there was substantial accumulation of CD68+ macrophages in mice with glomerulonephritis receiving (B) no treatment or (C) vehicle, which was reduced in (D) mice receiving BR-4628. Quantification of immunostaining of (E) glomerular CD68+ macrophages and (F) glomerular CD3+ T-cells show that there was a marked increase in the glomerular numbers of these cells in mice with glomerulonephritis receiving no treatment (No Tx) or vehicle, which was attenuated in mice receiving BR-4628. Furthermore, RT-PCR analysis showed that the development of glomerulonephritis resulted in significant increases in the kidney gene expression of (G) CD68, (H) CD3e, (I) CCL2, (J) TNF-α, (K) IFN-γ and (L) MMP-12 at day 15, which was reduced in mice receiving BR-4628. Magnification (A-D) x400. Graphed Data = mean ± SEM; n = 8. *p<0.05, **p<0.01, ***p<0.001.

Immunostaining identified a 5-fold increase in glomerular macrophages and a 3-fold increase in glomerular T-cells (3-fold) at day 15 of glomerulonephritis in untreated and vehicle-treated mice (Fig 5B–5F). BR-4628 treatment reduced the number of glomerular macrophages and T-cells by 30–40% in diseased kidneys, but did not affect kidney leukocyte numbers in normal mice (Fig 5D–5F).

Real-time PCR analysis of whole kidneys identified a marked increase in the gene expression of leukocyte markers (CD68, CD3e), proinflammatory cytokines (TNF-α, IFN-γ, CCL2) and macrophage elastase (MMP-12) in untreated and vehicle-treated mice at day 15 of glomerulonephritis (Fig 5G–5L). Treatment with BR-4628 reduced the gene expression of each of these markers of inflammation in mice with glomerulonephritis (Fig 5G–5L). In contrast, BR-4628 had no effect on basal expression of these genes in normal mice.

### BR-4628 does not affect tubular regulation of salt balance

**Fig 6:**
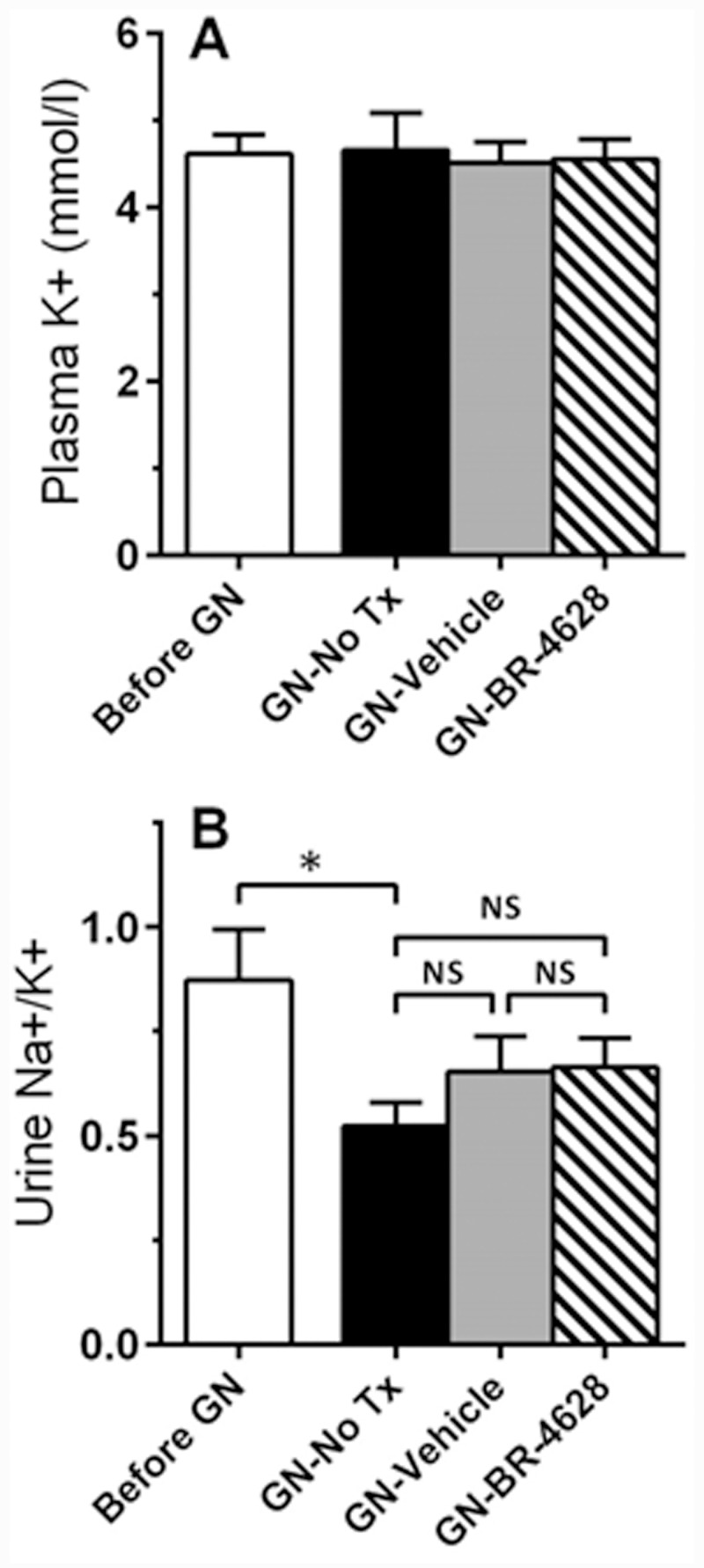
BR-4628 does not alter salt balance. (A) Plasma levels of K+ were similar in mice with and without glomerulonephritis, and were not affected by BR-4628 treatment. (B) After the development of glomerulonephritis there was slight reduction in urine levels of Na+/K+ in mice which received no treatment (No Tx), which was not altered by treatment with vehicle or BR-4628. Data = mean ± SEM; n = 8. *p<0.05, NS = not significant.

Plasma levels of K+ were similar in mice before and after induction of glomerulonephritis. Hyperkalemia was not seen in mice treated with BR-4628 (Fig 6A). Furthermore, assessment of urine levels of Na+ and K+ found that BR-4628 treatment had no effect on the Na+/K+ excretion rate in mice with glomerulonephritis (Fig 6B).

## Discussion

Our study is the first to demonstrate that a non-steroidal MRA (BR-4628) can reduce renal injury in a model of glomerulonephritis. In this model, BR-4628 inhibited glomerular crescent formation, accumulation of inflammatory cells and the loss of renal function. These pathological features of kidney disease have not previously been shown to be suppressed by a non-steroidal MRA. In addition, the protection obtained with BR-4628 was achieved without affecting tubular salt handling despite the presence of tubular damage, suggesting that this non-steroidal MRA may have distinct therapeutic benefits.

Glomerulonephritis is a major cause of chronic kidney disease. In this group of diseases, the primary injury occurs in the glomerulus which, if severe and persistent, can result in secondary damage to the tubulointerstitial compartment. In clinical studies, patients with chronic glomerulonephritis on standard RAS inhibitor therapy have increased protection against disease progression when a steroidal MRA is included in their treatment.[9,10] Adding a steroidal MRA to the intervention therapy reduces albuminuria and improves renal function in these patients, but can also lead to adverse side effects, such as an increased incidence of hyperkalemia, resulting in discontinuation of MRA use. In the current study, we found that treatment with a non-steroidal MRA (BR-4628) provided significant protection against declining renal function in a mouse model of glomerulonephritis, which was comparable to that previously achieved with a 20-fold higher dose of eplerenone in the same model.[5] In this study, BR-4628 therapy did not alter urine Na+/K+ excretion, whereas, eplerenone was shown to increase urine Na+/K+ excretion in this model.[5] Therefore, BR-4628 achieved similar protection to eplerenone in terms of clinical and histologic endpoints in this model of glomerulonephritis without causing tubular dysfunction in regards to salt handling. This suggests that BR-4628 treatment is potentially less likely to affect potassium homeostasis which can lead to hyperkalaemia.

Progressive forms of chronic kidney disease require inflammation to drive disease development. Treatment with steroidal MR antagonists has been shown to suppress the kidney expression of chemokines, proinflammatory cytokines and leukocyte adhesion molecules, and reduce kidney infiltration of leukocytes in animal models of glomerulonephritis and diabetic nephropathy.[4–6] Similarly, our study found that the protection afforded by BR-4628 in mouse glomerulonephritis was associated with reduced kidney accumulation of macrophages and T-cells and reduced gene expression of a macrophage chemoattractant (CCL2/MCP-1), proinflammatory cytokines (TNF-α, IFN-γ), and macrophage elastase (MMP-12) which can help facilitate macrophage migration through tissues. BR-4628 has also been shown to reduce kidney gene expression of chemokines (CCL2 and CXCL1) in a rat model of mineralocorticoid-dependent hypertension.[13] The importance of MR signaling in inflammatory cells has been recently demonstrated by a report showing that MR gene deletion in macrophages provides protection equivalent to that seen with steroidal MRA treatment in mouse glomerulonephritis, suggesting that MR-mediatated injury in this disease model is primarily due to macrophage MR signaling.[5] Interestingly, our study found that BR-4628 reduced kidney expression of IFN-γ which is known to increase MR expression in macrophages.[20] Therefore, BR-4628 may inhibit macrophage-mediated injury in glomerulonephritis by indirectly reducing MR levels in macrophages. What remains to be determined is whether BR-4628 can directly inhibit pathological responses in macrophages and whether it can do this more effectively than steroidal MR antagonists.

Rapidly progressive forms of glomerulonephritis usually exhibit severe histological damage (glomerular thrombosis, glomerulosclerosis, glomerular crescent formation and substantial tubular injury) in association with a rapid loss of renal function.[21] Our glomerulonephritis model displayed each of these features. Treatment of this model with BR-4628 significantly reduced the number of glomeruli with crescents, the severity of glomerular tuft lesions, tubular cell apoptosis, and the gene expression of markers of tubular injury (KIM-1) and fibrosis (collagen I, fibronectin, TGF-β1). BR-4628 has previously been shown to reduce KIM-1 mRNA levels in a model of mineralocorticoid-dependent hypertension in which renal damage and proteinuria are less severe.[13] Previous studies have also reported that steroidal MR antagonists can reduce glomerular crescent formation in models of anti-GBM glomerulonephritis and progressive lupus glomerulonephritis.[5,22] In addition, aldosterone is known to induce collagen and fibronectin expression by cultured mesangial cells and kidney fibroblasts, [23–26] and promote the *in vitro* proliferation of these cells, [27,28] suggesting that MRAs may directly inhibit fibrotic responses in the kidney. These studies indicate that MR signaling plays an important role in glomerular and tubular damage and fibrosis in glomerulonephritis and that MR antagonists, such as BR-4628, can effectively inhibit this MR-dependent injury, which may include direct effects on fibrotic responses.

Induction of anti-GBM glomerulonephritis results in the rapid development of podocyte injury and nephrotic range albuminuria.[5] Treatment with BR-4628 was unable to inhibit the development of albuminuria in mice with this disease. However, this outcome is not surprising, since previous experiments have shown that neither treatment with a steroidal MR antagonist nor MR gene deletion in podocytes were able to suppress albuminuria in this model.[5] These findings support the concept that podocyte injury and albuminuria are independent of MR signaling in this model. However, this does not rule out the possibility that BR-4628 treatment may succeed at reducing albuminuria in slower developing forms of glomerulonephritis or other chronic kidney diseases resulting from diabetes or hypertension, where steroidal MR antagonists are known to be effective.[3,6,29] Indeed, one previous study has shown that BR-4628 can inhibit proteinuria in a model of mineralocorticoid-induced hypertension.[13]

MR antagonists, including BR-4628, have been shown to reduce hypertension in animal models of disease, which can partially account for their protective effects in some disease settings.[13] In our study, we did not examine blood pressure in mice with anti-GBM glomerulonephritis. However, previous research has found that C57BL/6 mice do not develop hypertension in anti-GBM glomerulonephritis.[16] Furthermore, several animal studies have shown that steroidal MRAs can provide protection against renal injury at doses that do not affect blood pressure.[6–8] Therefore, the renal protection obtained with BR-4628 in our study is expected to be independent of any blood pressure effects.

Development of anti-GBM glomerulonephritis results from administration of an antibody that binds to the kidney glomerular basement membrane and initiates an immune response which involves activation of complement in glomeruli and subsequent recruitment of inflammatory cells. In this study, we did not examine the effect of BR-4628 on the humoral immune response because previous studies have shown that steroidal MR antagonists have no impact on the levels of circulating antibody or on the glomerular deposition of antibody or complement in this model.[3,5]

In conclusion, our study has established that the non-steroidal MR antagonist BR-4628 protects against development of glomerulonephritis without causing tubular dysfunction. This protection was primarily due to the inhibition of macrophage MR signaling which causes glomerular injury (inflammation, glomerular lesions, crescent formation) and subsequently promotes renal function impairment, tubular damage and the development of renal fibrosis. This study suggests that non-steroidal MR antagonists have future potential for the treatment of glomerulonephritis and other chronic inflammatory kidney diseases, such as diabetic nephropathy. A limitation of this study is that it demonstrates the effectiveness of BR-4628 in only a single model of glomerulonephritis which is rapidly progressive. There is a further need for non-steroidal MR antagonists to be assessed as intervention therapies in models of chronic kidney disease and to determine whether non-steroidal MR antagonists can provide additional protection when combined with current therapies in the treatment of patients with progressive forms of chronic kidney disease.

## Supporting Information

S1 Appendix **Table A**: qPCR probe and primer sequences.
(DOCX)

S2 Appendix NC3Rs ARRIVE Guidelines Checklist. (PDF)
