# Supplementary material for: Suppression of Rapidly Progressive Mouse Glomerulonephritis with the Non-Steroidal Mineralocorticoid Receptor Antagonist BR-4628
Source: PLoS One. 2015 Dec 23;10(12):e0145666. doi: 10.1371/journal.pone.0145666 (PMC4689384; doi:10.1371/journal.pone.0145666)
Supplement: S1 Appendix — Table A: qPCR probe and primer sequences. (DOCX) [file pone.0145666.s001.docx]

**S1 Appendix:**

**Table A**: qPCR probe and primer sequences

| **Target** | **Probe** | **Forward Primer** | **Reverse Primer** |
| --- | --- | --- | --- |
| **CD68** | CACAGTTTCTCCCACCA | CATGGGAATGCCACAATTTCT | ACAGTGGAGGATCTTGGACTA |
| **CD3e** | AGGAACCAGTGTAGAG | GCCGAGAACATTGAATACAAAG | TGGTGTGTAGCAGACGTAGTAG |
| **CCL2/MCP-1** | ACAACCACCTCAAGCAC | GACCCGTAAATCTGAAGCTAA | CACACTGGTCACTCCTACAGAA |
| **TNF-α** | TCACCCACACCGTCAG | GGCTGCCCCGACTACGT | TTTCTCCTGGTATGAGATAGCAATC |
| **IFN-γ** | CAACAGCAAGGCGAAA | CAGCAACAACATAAGCGTCA | ACCTCAAACTTGGCAATACTC |
| **KIM-1** | CAACAAGACCCACAAC | TTAAACCAGAGATTCCCACA | TTGGAGGAGTGGAGGTAGAGA |
| **Collagen I** | GTGGTGTGGTCGGTCT | GATCTCCTGGTGCTGATG | GAAGCCTCTTTCTCCTCTCTGA |
| **Fibronectin** | GGAGAGACAGGAGGAA | GCGCTATTACAGAATCACCTA | TTGATGGTGGCTGTGGACTT |
| **TGF-β1** | ACAACCAACACAACCC | GGACACACAGTACAGCAA | GACCCACGTAGTAGACGAT |
